# Kefir and the Gut–Skin Axis

**DOI:** 10.3390/ijerph192113791

**Published:** 2022-10-23

**Authors:** Emília Alves, João Gregório, Patrícia Rijo, Catarina Rosado, Luis Monteiro Rodrigues

**Affiliations:** 1 CBIOS—Universidade Lusófona’s Research Center for Biosciences & Health Technologies, Campo Grande 376, 1749-024 Lisboa, Portugal; 2 Health Sciences Ph.D. Program, University of Alcalá, Carretera Madrid-Barcelona, Km 33.100, 28805 Alcalá de Henares, Spain; 3 Instituto de Investigação do Medicamento (iMed.ULisboa), Faculdade de Farmácia, Universidade de Lisboa, 1649-003 Lisboa, Portugal

**Keywords:** kefir, intestinal health, skin health, gut microbiota, probiotics, gastrointestinal status, atopic dermatitis

## Abstract

The human gastrointestinal (GI) tract is a dynamic system influenced by various environmental factors, including diet and exposure to ingested probiotics, and prone to various functional impairments. These impairments are mostly related to any combination of motility alterations, visceral hypersensitivity, and changes in the mucosa, immune function, and intestinal microbiota. Intestinal microbial imbalance and immunological dysfunction have been linked to several chronic inflammatory disease states, including atopic dermatitis (AD). Disruption of the intestinal microbial balance, known as gut dysbiosis, has been demonstrated to negatively impact skin function by increasing the intestinal permeability. Consequently, the gut–skin axis may be receptive to modulation via dietary modification, namely, via ingestion of probiotics, thus representing interesting potential as an AD therapy. Kefir is an ancient probiotic food that has been demonstrated to positively impact the general condition of the digestive system, including the intestinal microbiota. However, the literature is still scarce on the impact on the gut–skin relationship of a diet containing kefir. This study, continuing research in our group, aimed to evaluate the impact of kefir intake on GI symptoms in healthy and AD skin subjects. Results showed a significant improvement in GI status, namely, in functional constipation, abdominal pain intensity, and abdominal distension, thus supporting the hypothesis that kefir intake is positively associated with improvement in GI status. The existence of a relationship between the improvement in skin parameters and the improvement in GI status after kefir consumption was established, thus reinforcing the role of homemade kefir as a potential modulator of the gut–skin axis in both healthy and atopic individuals.

## 1. Introduction

The gastrointestinal (GI) tract can be affected by a set of symptoms common to several organic pathologies and motility disorders, which include abdominal pain, diarrhea, constipation, bloating, fullness, nausea, and vomiting [1]. However, for a considerable number of individuals, these symptoms cannot be associated with any underlying structural abnormality, relying solely on the patient’s interpretation and description of their experience, thus being referred to as functional symptoms [1,2]. Visceral hypersensitivity, abnormal GI motility, and psychological disorders have long been recognized as contributors to the pathogenesis of these functional GI conditions. More recently, however, new factors have also been identified, such as low-grade intestinal inflammation, increased intestinal permeability, immune activation, and microbiome disturbances, thus calling into question the hypothesis that structural changes are entirely absent in functional disorders [1,3,4,5]. Although functional symptoms are often meal-related and can therefore vary, functional GI disorders are chronic conditions that share definable clinical features and can be characterized by standardized criteria [2,6]. The accepted criteria for diagnosing and classifying functional GI disorders were created and updated by the Rome Foundation, a committee of gastroenterologists and academics in the field of GI health, based on the consensus of expert opinion and with reference to currently available scientific evidence [2]. The assessment of the GI status of individuals should therefore take into account functional GI disorders defined as a group of GI symptoms related to any combination of motility disorders, visceral hypersensitivity, changes in the mucosa, immune function, and intestinal microbiota [1,3,5].

The intestinal microbiota can present itself in a state of balance, known as eubiosis, where the microbiota tolerates small changes resulting from the environment, diet, or water consumed, or in a state of imbalance, known as dysbiosis, resulting from changes such as the growth of specific bacterial groups, colonization by pathogenic bacteria, use of antibiotics, or major dietary changes [7,8]. Regardless of the state, the gut microbiota affects both physiological processes and other organs, such as the skin [7]. This relationship, known as the “gut–skin axis”, appears to be bidirectional [9,10,11], since the impairment of the gut microbiota is linked to the development of allergic diseases, where gut microbiota and/or dietary metabolites can be detected in the skin; skin health has been associated with intestinal barrier integrity and/or suppression of pro-inflammatory mediators [9,12,13].

The beneficial modulation of the intestinal microbiota is a current reality, whether due to technological advances or through foods, where probiotics play an essential role [14,15,16,17]. Foods with probiotic characteristics, typically resulting from fermentation processes, contain bioactive metabolites such as organic acids and short-chain fatty acids (SCFAs), which, in addition to being used as preferential sources of energy by intestinal cells, can modulate the host’s immune function, wherein the consumption of probiotics via fermented foods is associated with protection from metabolic and immune-mediated diseases [7,15,18]. The beneficial impact on host-microbiota interactions resulting from the use of probiotics may underlie a possible mechanism by which this type of food can positively impact human health, hence leading to the hypothesis of their potential action in the control of diseases associated with the intestinal microbiome, such as inflammatory diseases [9,10,11,15,19].

Kefir, one of the most ancient foods known to present health benefits commonly attributed to its microbial and nutritional richness, has already been demonstrated to positively impact the general condition of the digestive system, including the intestinal microbiota [16,20,21,22,23,24,25,26,27]. In addition to its increasing popularity, the nutritional and microbiological values of kefir make its application as a probiotic in the gut–skin axis regulation of the utmost interest. Regular consumption of kefir has been able to reduce gut dysbiosis, which opens the possibility that by modulation of the gut, inflammatory skin diseases may be better controlled [7,15,26,28]; however, to date, the literature is still scarce on the impact of a diet containing kefir on the gut–skin relationship.

Atopic dermatitis (AD) is a chronic inflammatory skin disease, but its pathogenesis appears to be associated not only with immune dysfunction but also with intestinal dysbiosis, thus evidencing a gut–skin relationship [10,11,29,30]. This intestinal dysbiosis has the ability to negatively impact skin function since it promotes immune dysregulation by increasing intestinal permeability via pro-inflammatory cytokines, thus contributing to chronic systemic inflammation [9,12,29]. Consequently, the gut–skin axis may be receptive to modulation via dietary modification, namely, via probiotic ingestion, thus representing a potential complementary alternative in AD therapy [31,32,33,34].

This study aimed to evaluate the impact of the ingestion of kefir on the GI status of both healthy and atopic skin individuals. This investigation gives continuity to previous studies by our group, where the relation between kefir intake and skin health improvement was found [35], and further supporting the evidence of a gut–skin axis in both atopic and healthy skin individuals.

## 2. Materials and Methods

### 2.1. Study Design

A controlled intervention study was conducted according to the principles of the Helsinki Declaration and after informed consent. The study protocol was approved by the ethics committee of the School of Sciences and Health Technologies at Lusofona’s University (Nº1/2018, 15 May 2018). Study subjects were recruited by convenience sampling between October 2019 and December 2020 among a university population (students and academic staff). Subjects were excluded from participating in the study according to the non-inclusion criteria (Supplementary Table S1). Only adult subjects were included and were assigned to different study groups according to their skin type, healthy and atopic. The atopic group (*n* = 19) included 1 male and 18 females, aged between 19 and 56 years (mean age 31.7 ± 11.9 years), wherein 47% were under 30 years old. Within this group, all subjects reported a clinical diagnosis of AD, 14 (74%) of the subjects also reported rhinitis, and 6 (32%) reported an asthma diagnosis. All other subjects who fulfilled the eligibility criteria, excluding the atopic criteria, and were free of skin diseases, including AD, psoriasis, and other systemic diseases that may impact skin conditions, were assigned to the healthy group. These subjects (*n* = 33) included 6 males (18%) and 27 females (82%), aged between 20 and 60 years (mean age 27.0 ± 10.1 years), wherein 61% were under 30 years old. Within the healthy (H) and the atopic (A) groups, volunteers were assigned to either the kefir intake (HK and AK, respectively) or the control (H0 and A0, respectively) group, according to their preference.

For each skin type evaluated, the effect of kefir ingestion between the intervention groups and respective controls was evaluated using a parallel group design. However, in order to minimize baseline individual variability, especially in studies involving dietary interventions, a crossover design was also sequentially applied to each study group, where individuals were used as their own controls (paired comparisons).

All subjects were instructed to avoid excessive exercise, consumption of food supplements or fermented foods, use of laxatives and change their usual food intake. Moreover, avoid changes in the type and frequency of skin care agents used regularly and traveling abroad during the study period.

The clinical endpoint in this study was the evaluation of the GI status after kefir intake.

#### Baseline Conditions

Physiological, sociodemographic, and lifestyle conditions, as well as dietary intake profiles were assessed, at baseline, before the beginning of the intervention, as described by the authors elsewhere [35].

### 2.2. Kefir Intervention

The intervention consisted of the daily consumption of kefir during eight weeks, which is the time range accepted in the literature for this type of intervention in similar trials [36,37,38,39,40]. The kefir was produced by fermentation of a commercial ultra-high temperature pasteurized (UHT) semi-skimmed cow milk of Portuguese provenance (Nova Açores^®^, S. Miguel, Portugal), with CIDCA AGK1 kefir grains, obtained from the Centro de Investigación y Desarrollo en Criotecnología de Alimentos (CIDCA), La Plata, Argentina, using a grain inoculum of 10% (*w*/*v*), for 24 h, at a temperature of 20 ± 1 °C, replicating Portuguese household representative conditions [41]. Prior to the intervention, the kefir beverage consumed during this study was characterized by the authors, including the description of the preparation, storage, and intake conditions [42].

### 2.3. Skin Measurements

Skin conditions were assessed by measuring transepidermal water loss (TEWL) and stratum corneum (SC) hydration for all participants; for the atopic skin group, an additional assessment of AD clinical severity was performed using the standard atopic dermatitis—SCORAD scoring system [43,44], as previously described by the authors [35].

### 2.4. GI Status Assessment

**Figure 1:**
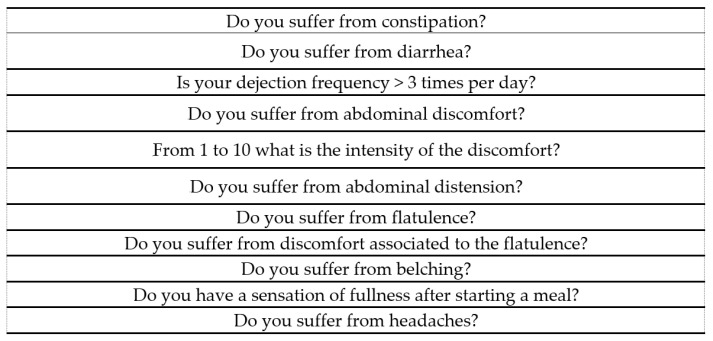
Gastrointestinal status questionnaire.

Participants’ GI status was assessed at baseline (t0) and the end of the eight-week intervention period (t8) using a self-completed questionnaire, adapted from the recommendations of the Rome Foundation [4] (Figure 1). Each parameter was coded as follows: yes = 2, sometimes = 1 and never = 0. The outcome “Improved GI status” was considered when variable at t8 <variable at t0.

### 2.5. Statistical Analysis

Results were expressed as relative frequencies. Since the data were not normally distributed (normality assessed by the Shapiro–Wilk test), non-parametric tests were chosen to test different hypotheses. Differences within individuals were identified by Wilcoxon signed rank test and differences between kefir intake and control groups by Chi-Square test. Logistic regressions were used to evaluate the association between kefir intake and GI status improvements and their potential confounding factors. All analyses were performed using the SPSS statistical package version 25 (SPSS Inc., Chicago, IL, USA) with a significance level of 0.05.

## 3. Results

Neither healthy nor atopic subjects who were given kefir showed any differences in baseline characteristics, either physiological, sociodemographic, and regarding dietary intake, when compared to the respective control groups, as previously described by the authors [35].

### 3.1. GI Status Assessment

At baseline, no differences in GI status were observed between the kefir intake groups and the respective controls, for both healthy and atopic groups (*p* > 0.05, for all parameters, Supplemental Table S2).

The variation in GI status was analyzed after eight weeks of intervention, comparing the intake of the kefir groups and the respective controls, in both healthy and atopic skin volunteers (Table 1). Comparisons were made using the outcome variable “Improved GI status”.

As shown in Table 1, data revealed that in healthy skin subjects, kefir ingestion for eight weeks improved functional constipation (*p* = 0.003), functional diarrhea as well as dejection frequency (*p* = 0.008 and *p* = 0.024, respectively), intensity of abdominal discomfort (*p* = 0.008), functional abdominal distension *(p* < 0.001), discomfort associated with flatulence (*p* = 0.024) and headache (*p* = 0.008), when compared to control.

Furthermore, because GI status can also be influenced by intrinsic individual variation, an individual paired comparison, between t0 and t8, was performed. The results obtained support the data shown in Table 1 since it was observed that healthy skin individuals who drank kefir for eight weeks significantly improved functional constipation, functional diarrhea, functional abdominal distension, and flatulence *(p* = 0.025, *p* = 0.046, *p* = 0.008, and *p* = 0.025, respectively) after that period, while no differences were found after this period in those who did not drink kefir (*p* > 0.05, for all GI status) (Supplemental Table S3).

A similar analysis regarding atopic subjects revealed significant improvements in functional constipation and functional abdominal distension *(p* = 0.033 and *p* = 0.040, respectively) for the kefir intake group compared to the control (Table 1). These results were also reinforced by those from individual paired comparisons that showed that at t8, the atopic subjects who drank kefir presented a significant improvement in functional constipation, functional abdominal distension, and discomfort associated with flatulence (*p* = 0.038, *p* = 0.023, and *p* = 0.015, respectively). In controls, no differences were observed after the intervention period (*p* > 0.05, for all GI statuses) (Supplemental Table S3).

### 3.2. Adjusted Models for GI Status

To assess the effect of different independent variables on the outcomes of GI status, logistic regression models were performed. All socio-demographic variables, food intake variables, kefir intake, and skin status were considered as possible predictors for the influence in the variation of the GI status outcomes. After testing the assumptions for collinearity diagnostics, independent variables were excluded from the models if the variance inflation factor (VIF) was superior to 10. After this step, logistic regressions were performed for each outcome variable to determine possible predictor variables for GI status outcomes. The most common variables in the models identified by this method were—kefir status, defined as with or without kefir intake, water intake in liters, and age, defined as less than 30 years old or greater than or equal to 30 years old. Final logistic regressions were then run and are presented in Table 2.

Results from Table 2 showed that drinking kefir for eight weeks is associated with a significant improvement in functional constipation, the intensity of abdominal discomfort, abdominal distension, and headache (crude OR). In the model adjusted for water intake (aOR1), the effect of kefir intake remained significant for the same symptoms, with an additional significant improvement for belching. Further adjustments for age (aOR2) showed that the effect of kefir intake remained significant for the previous symptoms, with an additional significant improvement for flatulence frequency (*p* = 0.040).

These results showed that kefir intake was positively associated with GI status improvement, particularly for functional constipation, the intensity of abdominal discomfort, functional abdominal distension, and headache, with the water intake having a higher impact in all of the above as well as in belching, as expected due to the established beneficial impact of water intake in the gut [34,45]. Functional abdominal distention and belching have been shown to be more common in those over 30 years old, which may be partially explained by the physiological aging of the GI system [46].

### 3.3. GI Status and Modification of Skin Parameters

This study aimed to explore potential relationships between the gut and the skin, assessed by improvement of skin barrier condition and GI status. The set of results related to the skin, obtained by the authors in a previous study, showed that both healthy and atopic skin subjects who ingested kefir for eight weeks (*n* = 22) showed improvement in the following skin parameters: TEWL of the forearm and forehead and hydration of the forearm, with individuals with atopic skin also showing improvement in the SCORAD index [35]. Taking this into account and in light of the results obtained in this study (Table 2), a more in-depth analysis of the existence of a relationship between the improvement of skin parameters and the improvement of the GI status for those who drank kefir was conducted (Table 3). As no differences were observed in the control groups either in the skin parameters [35] or in the GI status (Supplementary Table S3), these groups were not considered for this analysis.

As shown in Table 3, from the 22 individuals that showed skin improvement after eight weeks of kefir ingestion, 22.7% (*n* = 5) of those who showed improvement in forearm hydration also reported a significant improvement in functional diarrhea (*p* = 0.038). In addition, 45.5% (*n* = 10) of the individuals who showed improvement in forehead TEWL also showed significant improvement in discomfort associated with flatulence (*p* = 0.041). Furthermore, in more than 50% of the atopic subjects that showed a decrease in the SCORAD index, improvements were reported in terms of functional constipation, the intensity of abdominal discomfort, abdominal distension, and discomfort associated with flatulence, although the results were not statistically significant.

## 4. Discussion

In this study, in healthy skin volunteers, drinking kefir for eight weeks was associated with a significant improvement in GI status, namely, in functional constipation, the intensity of abdominal discomfort, functional abdominal distension, fullness sensation, headache, and flatulence frequency (Table 1). Similar results were found in other studies in humans regarding the effect of kefir consumption on GI functionality [24,25,47,48]. Maki et al., studying patients with constipation, observed that lyophilized kefir had no impact on laxative use, stool consistency, and stool volume compared to control; however, the number of patients not requiring any laxatives was higher twelve weeks following the kefir intervention compared to baseline [25]. Turan et al., showed that taking kefir for four weeks significantly increased stool frequency, improved bowel satisfaction score, and reduced gut transit time compared to baseline in people with functional constipation [48]. Hertzler et al., observed a significant decrease in flatulence severity after a five-day kefir intervention in people with lactose malabsorption, but no differences were found for flatulence frequency, abdominal pain, or diarrhea [47]. Bekar et al., investigated the impact of kefir on *Helicobacter pylori* eradication rates in patients with dyspepsia and found a significantly higher rate of eradication in the kefir group compared to the control group, accompanied by a significantly lower occurrence of diarrhea, abdominal pain, and nausea compared to control [24].

In our study, in subjects with atopic skin, improvements were evident only in the kefir intake group and were restricted to functional constipation and abdominal distension (Table 1). Notably, none of the in vivo human studies found in the literature concerning inflammatory skin diseases, such as AD, observed the impact of a diet containing traditionally homemade kefir as the probiotic, on both the skin and the GI status. However, studies assessing the effect of kefir on the gut of individuals with pathologies associated with low-grade inflammation support kefir’s ability to positively impact both the gut and inflammation [14,18,49]. Bellikci-Koyu et al., evaluating the intestinal impact of kefir consumption in patients with metabolic syndrome, observed a significant positive change in the gut microbiota that was correlated with an improvement in metabolic syndrome conditions, thus supporting the kefir’s positive impact on inflammatory diseases through the gut [14]. Praznikar et al., assessing the effect of kefir intake in overweight adults, found an improvement in serum zonulin levels, an important intestinal barrier dysfunction marker, which enhanced the kefir probiotic capacity to modulate intestinal microbiota composition and consequent capacity to control the grade of the chronic inflammation [18]. St-Onge et al., testing the impact of kefir in hypercholesterolemic subjects, found a significant increase in fecal SCFAs, which was evidence of the positive effect of kefir on intestinal regulation [49].

The differences in the improvement of GI status observed in this study between subjects with healthy and atopic skin may be partially explained by the presence of intestinal dysbiosis that characterizes patients with AD [30,50], since, for these, a longer intervention period may be necessary to detect further improvements in GI status, particularly if assessed solely by self-reported symptoms [12].

The existence of a relationship between kefir intake and the improvement in both skin parameters and in GI status demonstrated in this work is in line with the recent work of Fang and coworkers who found that the intake of a strain-specific probiotic (*Lactobacillus* and *Bifidobacterium* species) for eight weeks ameliorated severity in AD subjects through the improvement of gut health, thus supporting the hypothesis that the ingestion of probiotics promotes recovery from a state of intestinal dysbiosis to a state of healthy balance [51]. Identical results were found by Kim et al., in a previous meta-analysis of adults with AD, which showed significant differences in SCORAD values favoring strain-specific probiotics over controls [52]. In a recent systematic review investigating the efficacy of probiotics on the severity of AD, Petersen et al., verified that of fifteen studies that showed alterations in the gut microbial composition promoted by the use of probiotics, only eight were able to link those alterations to a positive effect on the severity of AD [30]. The study had several limitations, however, including the degree of AD severity at baseline among the studies reviewed.

Some limitations must be acknowledged (i) a double-blind, placebo-controlled study would be recommended (although difficult to implement), and the small dimension of groups made it difficult to detect small to medium shifts in the gut environment (*β*-error) [53]; (ii) the use of a non-validated questionnaire to collect self-reported symptoms is prone to biases [54,55] and, (iii) the composition of the human gut microbiome was not monitored [56]. Additional studies are necessary to understand the mechanisms underlying the modulation of the gut–skin axis.

## 5. Conclusions

To the best of our knowledge, this was the first study to provide information on the impact of kefir intake, produced under household conditions, on the GI status in healthy and atopic skin individuals. The improvement in GI status in atopic individuals is a novel observation and confirms the gut–skin axis connection.

This work supports the hypothesis that the ingestion of probiotics in fermented foods improves both skin and GI conditions.

## Figures and Tables

**Table 1:** GI status improvement, after eight weeks of kefir ingestion, between kefir intake and control groups, for both healthy and atopic skin volunteers (relative frequency (%)).

Improved GI Status	Healthy Group (*n* = 33)			Atopic Group (*n* = 19)		
	HK	H0	*p*-Value	AK	A0	*p*-Value
Functional constipation, % (*n*)	38.5 (5)	0.0 (0)	**0.003**	55.6 (5)	10.0 (1)	**0.033**
Functional diarrhea, % (*n*)	30.8 (4)	0.0 (0)	**0.008**	11.1 (1)	20.0 (2)	0.596
Dejection frequency > 3 times per day, % (*n*)	23.1 (3)	0.0 (0)	**0.024**	22.2 (2)	20.0 (2)	0.906
Intensity of abdominal discomfort ≥ 5, % (*n*)	30.8 (4)	0.0 (0)	**0.008**	55.6 (5)	30.0 (3)	0.260
Functional abdominal distension, % (*n*)	53.8 (7)	0.0 (0)	**<0.001**	66.7 (6)	20.0 (2)	**0.040**
Flatulence frequency, % (*n*)	38.5 (5)	10.0 (2)	0.051	55.6 (5)	40.0 (4)	0.498
Associated discomfort, % (*n*)	23.1 (3)	0.0 (0)	**0.024**	77.8 (7)	60.0 (6)	0.405
Belching, % (*n*)	15.4 (2)	0.0 (0)	0.070	44.4 (4)	20.0 (2)	0.252
Fullness sensation, % (*n*)	n.a.	n.a.	--	22.2 (2)	30.0 (3)	0.701
Headache, % (*n*)	30.8 (4)	0	**0.008**	33.3 (3)	20.0 (2)	0.510

HK—healthy skin with kefir intake; H0—healthy skin without kefir intake; AK—atopic skin with kefir intake; A0—atopic skin without kefir intake. Groups were compared by chi-square test, *p* < 0.05 for statistical significance. n.a.—not applicable because outcome is constant.

**Table 2:** Association between kefir intake and GI status improvement (Odds Ratio (*p*-value), *n* = 52).

Improved GI Status	Odds Ratio (*p*-Value)		
	Crude OR	aOR1	aOR2
Functional constipation	24.17 (**0.004**)	33.93 (**0.003**)	32.22 (**0.003**)
Functional diarrhea	4.118 (0.112)	3.901 (0.138)	4.150 (0.128)
Dejection frequency > 3 times per day	4.118 (0.112)	3.869 (0.137)	3.868 (0.137)
Intensity of abdominal discomfort ≥ 5	6.231 (**0.014**)	6.208 (**0.015**)	6.153 (**0.016**)
Functional abdominal distension	20.22 (**<0.001**)	27.74 (**<0.001**)	30.29 (**<0.001**)
Flatulence frequency	3.333 (0.054)	3.326 (0.056)	3.994 (**0.040**)
Associated discomfort	3.333 (0.054)	3.321 (0.056)	3.446 (0.052)
Belching	5.250 (0.058)	6.103 (**0.046**)	8.125 (**0.033**)
Fullness sensation	0.900 (0.913)	0.920 (0.931)	0.836 (0.854)
Headache	6.533 (**0.030**)	6.719 (**0.028**)	6.635 (**0.031**)

OR—odds ratio for kefir status (Reference category: without kefir intake (*n* = 33)); aOR1—odds ratio for kefir status adjusted for water intake; aOR2—odds ratio for kefir status adjusted for water intake and cut-off age 30 years old, *p* < 0.05 for statistical significance.

**Table 3:** Comparison between GI status improvement and skin parameters modification, after eight weeks of kefir intake (relative frequency (%) (*p*-value), *n* = 22).

Modification of Skin Parameters	Improved GI Status					
	Functional Constipation	Functional Diarrhea	Dejection Frequency > 3 Times per Day	Intensity of Abdominal Discomfort ≥ 5	Functional Abdominal Distension	Discomfort Associated to Flatulence
TEWL, *n* (%)						
Forearm	10 (45.4%) (0.644)	5 (22.7%) (0.557)	5 (22.7%) (0.906)	9 (40.9%) (0.301)	13 (59.1 %) (0.271)	10 (45.4 %) (0.235)
Forehead	10 (45.4%) (0.510)	5 (22.7%) (0.327)	5 (22.7%) (0.225)	9 (40.9%) (0.271)	13 (59.1 %) (0.815)	10 (45.4 %) (**0.041**)
Forearm Hydration, *n* (%)	10 (45.4%) (0.668)	5 (22.7%) (**0.038**)	5 (22.7%) (0.196)	9 (40.9%) (0.894)	13 (59.1 %) (0.229)	10 (45.4 %) (0.070)
SCORAD Index ^O^, *n* (%)	5 (55.6%) (1.000)	1 (11.1%) (0.439)	2 (22.2%) (0.558)	5 (55.6%) (0.327)	6 (66.7%) (0.197)	7 (77.8%) (0.380)

TEWL—transepidermal water loss. SCORAD—scoring of atopic dermatitis. **^O^**—atopics only (*n* = 9). Groups compared by Mann–Whitney U-test, with *p* < 0.05 for statistical significance.

## Data Availability

Data are available from the corresponding author upon reasonable request.

## Supplementary Materials

The following are available online at https://www.mdpi.com/article/10.3390/ijerph192113791/s1, Table S1: Non-inclusion criteria. Table S2: Baseline GI status parameters for each study group. Table S3: Individual variation in GI status, between t8 and t0.
