# Supplementary material for: Kefir and the Gut–Skin Axis
Source: Int J Environ Res Public Health. 2022 Oct 23;19(21):13791. doi: 10.3390/ijerph192113791 (PMC9653948; doi:10.3390/ijerph192113791)
Supplement: Supplementary file 1 [file ijerph-19-13791-s001.zip › ijerph-1955535-supplementary.pdf]

International Journal of
Environmental Research and Public Health

MDPI

Supplementary Material

Supplemental Table S1 – Non-inclusion criteria.

Non-inclusion criteria
1. Regular consumption of kefir or any probiotic strains (as supplements or pharmaceuticals) in the 3 months prior to the study or during the study<br>2. Oncologic disease<br>3. Women pregnant or breastfeeding<br>4. Retinoid treatment in the 3 months prior to the study or during the study<br>5. Antibiotic treatment in the 30 days prior to the study or during the study<br>6. Topical treatment with corticosteroids / anti-inflammatories in the study area in the 8 days prior to the study or during the study<br>7. Chronic illness involving taking regular (daily) medications such as insulin, oral antidiabetics, anti-inflammatories or immunosuppressants<br>8. Skin disease in the study areas<br>9. Cosmetic treatment on the skin, scrubbing, or depilation at the study areas in the 30 days, or during the study period<br>10. Failure to comply with the guidelines of the study

Supplemental Table S2 – Baseline GI status parameters for each study group (relative frequency).

Baseline GI Status	Healthy Group (n = 33)			Atopic Group (n = 19)		
	HK	H0	*p*-Value	AK	A0	*p*-Value
Functional constipation, % (n)	38.5 (5)	25.0 (5)	0.076	55.6 (5)	60.0 (6)	0.956
Functional diarrhea, % (n)	30.8 (4)	25.0 (5)	0.452	22.2 (2)	40.0 (4)	0.405
Dejection frequency > 3 times per day, % (n)	30.8 (4)	25.0 (5)	0.178	22.2 (2)	20.0 (2)	0.362
Intensity of abdominal discomfort ≥ 5, % (n)	23.1 (3)	5.0 (1)	0.438	66.7 (6)	60.0 (6)	0.634
Functional abdominal distension, % (n)	53.8 (7)	55.0 (11)	0.449	88.9 (8)	90.0 (9)	0.242
Flatulence frequency, % (n)	61.5 (8)	55.0 (11)	0.832	88.9 (8)	90.0 (9)	0.782
Associated discomfort, % (n)	30.8 (4)	35.0 (7)	0.136	88.9 (8)	70.0 (7)	0.563
Belching, % (n)	15.4 (2)	25.0 (5)	0.784	55.6 (5)	30.0 (3)	0.413
Fullness sensation, % (n)	30.8 (4)	25.0 (5)	0.716	33.3 (3)	50.0 (5)	0.312
Headache, % (n)	92.3 (12)	65.0 (13)	0.076	55.6 (5)	70.0 (7)	0.445

Groups were compared by Chi-Square test, with $p < 0.05$ for statistical significance. n.a.- not aplicable, outcome is constant.

Supplemental Table S3 – Individual variation in GI status, between t8 and t0 (Wilcoxon Standartized (Z) test statistic (p-value)).

GI STATUS	Healthy Group (n = 33)		Atopic Group (n = 19)	
	**HK**	**H0**	**AK**	**A0**
Functional constipation	-2.236 (0.025)	0.000 (1.000)	-2.707 (**0.038**)	-1.000 (0.317)
Functional diarrhea	-2.000 (**0.046**)	0.000 (1.000)	-1.000 (0.317)	-1.414 (0.157)
Dejection frequency > 3 times per day	-1.633 (0.102)	0.000 (1.000)	-1.342 (0.180)	-1.414 (0.157)
Intensity of abdominal discomfort ≥ 5	-1.841 (0.066)	0.000 (1.000)	-1.612 (0.107)	-1.214 (0.225)
Functional abdominal distension	-2.646 (**0.008**)	0.000 (1.000)	-2.271 (**0.023**)	-0.577 (0.564)
Flatulence frequency	-2.236 (**0.025**)	-1.414 (0.157)	-1.947 (0.052)	-0.333 (0.739)
Associated discomfort	-1.633 (0.102)	0.000 (1.000)	-2.428 (**0.015**)	-1.265 (0.206)
Belching	-1.414 (0.157)	0.000 (1.000)	-1.890 (0.059)	-1.342 (0.180)
Fullness sensation	0.000 (1.000)	0.000 (1.000)	-1.342 (0.180)	-0.447 (0.655)
Headache	-1.857 (0.063)	0.000 (1.000)	-1.633 (0.102)	-0.447 (0.655) ¥

HK – Healthy skin with kefir intake; H0 - Healthy skin without kefir intake; AK – Atopic skin with kefir intake; A0 – Atopic skin without kefir intake. Individuals were compared by Wilcoxon signed rank test, with $p<0.05$ for statistical significance. ¥ - based on negative ranks (variable at t0 < variable at t8).
